# Case Report of an Adolescent with Major Depressive Disorder and Recurrent Suicide Attempts

**DOI:** 10.1192/j.eurpsy.2026.11151

**Published:** 2026-07-31

**Authors:** S. E. Ilgın, S. Göktaş, Y. Arıdaşır

**Affiliations:** 1 Psychiatry, Marmara University Pendik Research and Education Hospital; 2 Psychology, Yeditepe University; 3 Child and adolescent psychiatry, Marmara University Pendik Research and Education Hospital, İstanbul, Türkiye

## Abstract

**Introduction:**

Major depression is a type of mood disorder that develops following genetic, environmental, or hormonal disturbances. Major depressive disorder is the psychiatric condition with the highest rates of suicide. Although suicide ranks lower among causes of death in adults, it is the third leading cause of death among children and adolescents aged 10–24. Additionally, uncontrolled and excessive use of the internet, especially during the critical period of adolescence, can lead to health problems such as obesity.

**Objectives:**

This case report examines the interplay between major depressive disorder, social anxiety disorder, gaming addiction, and morbid obesity in an adolescent, focusing on psychosocial and familial factors, and management of suicidal ideation.

**Methods:**

The patient’s psychosocial and psychiatric status was assessed through clinical observation, family interviews, and psychometric tests including the Young Internet Addiction Scale, KADS-11, Social Anxiety Scale for Children, and Beck Hopelessness Scale. Pharmacological treatment was provided.

**Results:**

Z.A. lives in Istanbul, Turkey, in a family where the mother has depressive mood and anger problems, the father has physical and mild intellectual disabilities, and the older brother is unemployed. Z.A. is morbidly obese with no other medical conditions and is being followed for major depressive disorder, social anxiety disorder, and gaming addiction. Current medications include sertraline 15 mg, escitalopram 20 mg, methylphenidate 54 mg, and aripiprazole 20 mg, with risperidone 5 mg as needed. The patient has a history of multiple suicide attempts, most recently a wrist-cutting incident in June 2025. Z.A. experiences intense social anxiety, spends much of the time gaming, eating, and sleeping, and develops self-harm thoughts for relief. Psychometric assessments showed risky internet use (Young Internet Addiction Scale: 57), severe depression (KADS-11: 25), high social anxiety (Social Anxiety Scale for Children: 79), and severe hopelessness (Beck Hopelessness Scale: 20/20). Following an increase in suicidal thoughts, Z.A. was placed under weekly follow-up, and hospitalization was decided on September 4, 2025, based on family reports.

**Conclusions:**

Z.A.’s risky internet use, combined with social anxiety, reinforces social isolation, exacerbating psychological symptoms while contributing to a sedentary lifestyle and increased body mass index. This situation strengthens the negative cycle in which both depression and morbid obesity reinforce each other, posing additional risks to physical health and psychological well-being. Family factors also play a significant role in the patient’s psychopathology. The mother’s depressive mood and anger issues, along with the father’s physical and mild intellectual disabilities, may limit Z.A.’s support system and reduce stress tolerance.

**Disclosure of Interest:**

None Declared

